# Noise Propagation in Gene Regulation Networks Involving Interlinked Positive and Negative Feedback Loops

**DOI:** 10.1371/journal.pone.0051840

**Published:** 2012-12-20

**Authors:** Hui Zhang, Yueling Chen, Yong Chen

**Affiliations:** 1 Institute of Theoretical Physics, Lanzhou University, Lanzhou, China; 2 Department of Physics, Gansu College of Traditional Chinese Medicine, Lanzhou, China; 3 Department of Mathematics, Kings College London, London, United Kingdom; Keio University, Japan

## Abstract

It is well known that noise is inevitable in gene regulatory networks due to the low-copy numbers of molecules and local environmental fluctuations. The prediction of noise effects is a key issue in ensuring reliable transmission of information. Interlinked positive and negative feedback loops are essential signal transduction motifs in biological networks. Positive feedback loops are generally believed to induce a switch-like behavior, whereas negative feedback loops are thought to suppress noise effects. Here, by using the signal sensitivity (susceptibility) and noise amplification to quantify noise propagation, we analyze an abstract model of the Myc/E2F/MiR-17-92 network that is composed of a coupling between the E2F/Myc positive feedback loop and the E2F/Myc/miR-17-92 negative feedback loop. The role of the feedback loop on noise effects is found to depend on the dynamic properties of the system. When the system is in monostability or bistability with high protein concentrations, noise is consistently suppressed. However, the negative feedback loop reduces this suppression ability (or improves the noise propagation) and enhances signal sensitivity. In the case of excitability, bistability, or monostability, noise is enhanced at low protein concentrations. The negative feedback loop reduces this noise enhancement as well as the signal sensitivity. In all cases, the positive feedback loop acts contrary to the negative feedback loop. We also found that increasing the time scale of the protein module or decreasing the noise autocorrelation time can enhance noise suppression; however, the systems sensitivity remains unchanged. Taken together, our results suggest that the negative/positive feedback mechanisms in coupled feedback loop dynamically buffer noise effects rather than only suppressing or amplifying the noise.

## Introduction

Gene expression is a complex stochastic process involving numerous components and reaction steps. It also spans several time and concentration scales, including gene transcription, translation, and chromosome remodeling. Ozbuda et al. provided the first direct experimental evidence of the biochemical origin of phenotypic noise, this concept is currently receiving increased attention [1]. Phenotypic noise is due to low-copy-number molecules and fluctuations in the local environment [2]. Recently, a quantitative model of noise in genetic networks has been established, and the components that contribute to fluctuations have been suggested [3]. Noise has been found to play a pivotal role in phenotypic variation and cellular differentiation [4]. Fluctuations can be considered useful for balancing precision and diversity in eukaryotic gene expression [5] and for promoting non-genetic diversity to increase the survival capabilities of prokaryotic gene expression [6]. More importantly, noise affects most biological processes, such as cellular development and the determination of cellular fates [7]. Noise levels are also related to the frequency of cellular differentiation, and a noise-related motif can be adjusted based on its dynamic behavior [8].

In a living cell, a gene regulatory network is a complex web in which a reliable signal must be propagated from one gene to the next to execute its genetic program. However, the stochastic fluctuations in gene expression and expression fluctuations transmitted from one gene to the next may interfere with genetic programs [9], and there are inherent noises in the cellular signal transduction networks [10]–[12]. Therefore, expression of a gene can be affected by fluctuations upstream. Thus, a gene's noise can be propagated from its upstream genes. Thus, it is important to address the interactions between gene expression and noise in regulatory networks.

It is well known that, in a gene regulatory network, feedback loops are considered to be common [13] and have a critical role in cellular signaling networks [14], [15]. A feedback loop can use its output as an input signal to adapt to external and internal changes [16]. Hooshangi et al. suggested that the prevalence of feedback motifs in natural systems can be attributed to other favorable cellular behaviors and may provide robustness to extrinsic noise [17]. In addition, Passos et al. showed that a feedback loop is both necessary and sufficient for the stability of growth arrest during the establishment of a senescent phenotype [18]. Moreover, a large number of positive and negative feedback loops exist in various gene regulatory networks. A negative feedback loop produces system stability in genetic and biochemical networks [19] and induces the oscillatory [20] and linear dose-response relationship in gene expression [21]. The positive feedback loop in the mitogen-activated protein kinase (MAPK) cascade of Xenopus oocytes can produce a switch-like response [22], [23] and improves cellular memory [24]. Positive feedback loops can also suppress noise propagation [25]. Both negative and positive feedback loops have important implications for controlling noise in gene regulatory networks.

**Figure 1:**
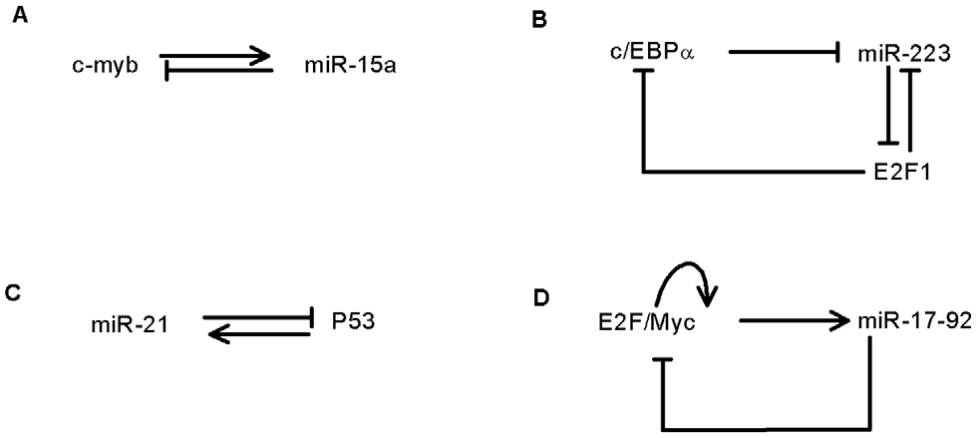
Examples of the interlinked positive and negative feedback loops in genetic networks. (A) The interaction between C-myb and miR-15a in human hematopoietic cells. (B) The regulation between cell cycle E2F1 and mizr-223 in acute myeloid leukemia. (C) MiR-21 targets p53 in Glioblastoma cells, and (D) the feedback loops involving miR-17-92, E2F and Myc in cancer networks.

Interestingly, interlinked positive and negative feedback loops are favored by biological systems. Single positive/negative feedback loops are also important [26], [27]. Examples include the interaction between miR-15a and c-myb in human hematopoietic cells [28], the regulation between cell cycle E2F1 and miR-223 in acute myeloid leukemia [29], the p53-targeting of miR-21 in Glioblastoma cells [30], and the feedback loops involving miR-17-92, E2F and Myc in cancer networks [31] (see Figure 1). Tian et al. suggested that a combination of positive and negative feedback loops can fine-tune dynamic behaviors in gene regulatory networks [28] and create flexible biological switches [32], [33]. Moreover, Hornung showed that fast negative feedback loops can enhance the system stability and that positive feedback can buffer noise propagation within the network via a combination of positive and negative feedback loops [25]. However, it is still not clear how interlinked positive and negative feedback loops affect signal propagation in gene regulatory networks, or how they act in response to stochastic fluctuations in gene expression.

To answer these questions, we developed a formula with plausible experimental parameters that represents the systemic signal sensitivity and noise amplification in an abstracted model of E2F/Myc/miR-17-92 networks. Our results show that the role of feedback loop in sensitivity and noise amplification is related to the dynamic properties of the system. The noise is always buffered at high protein concentrations in the monostable and bistable regions, but this buffering ability is reduced by the existence of negative feedback. While the signal sensitivity is enhanced in these regions, the improvements in noise propagation are due to negative feedback. Additionally, the noise is constantly enhanced at low protein concentrations in the excitable, bistable and monostable regions, but this enhancement ability is reduced by the existence of negative feedback. The signal sensitivity is also reduced in these regions. Notably, the effect of positive feedback is opposite to that of negative feedback. Furthermore, we also found that a fast-reacting protein module or a low noise autocorrelation time can enhance noise suppression without changing the signal sensitivity of the system. Our findings show that interlinked positive and negative feedback loops play a key role in reliable signal propagation.

## Results

### Model formulation

**Figure 2:**
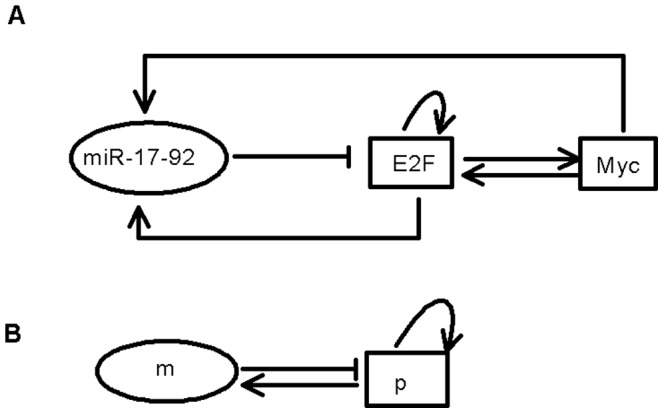
An illustration of the reducing process in cancer networks involving miR-17-92, E2F, and Myc. (A) Model of the interaction between E2F, Myc and miR-17-92. (B) The final reduced abstract model. Variables 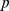 and 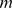 represent the protein module (Myc and E2Fs) and the miR-17-92 gene cluster, respectively.

In this study, we use the abstract model of miR-17-92 and E2F/Myc that was presented by Aguda et al. Figure 2 illustrates the essential abstract structure of the Myc/E2F/miR-17-92 network [31], [34]. The terms 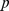 and 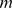 are used to describe the protein module (Myc and E2F) and miRNA cluster module, respectively. The basic principles of the network can be described in terms of 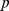 and 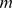 in the following equations,
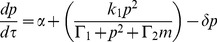
(1)

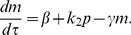
(2)Here, 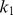 represents the protein module's autocatalytic process, which is a positive feedback loop, and 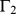 represents the protein that is inhibited by the miRNAs. At the same time, protein module 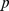 induces the transcription of miRNAs 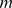, which is denoted by 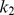. The protein is eliminated with a rate constant of 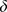. The number of the proteins stimulated by a growth factor synthesized in the extracellular medium is 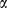. The constant 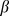 in Eq. (2) represents the transcription of 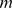, and 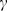 is the degradation rate of 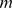.

Using the dimensionless variables and parameters, 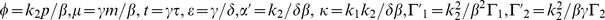, Eqs. (1) and (2) can be rewritten as follows by a series of nondimensionalizing processes [31],
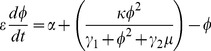
(3)

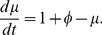
(4)Because that protein is less stable than miRNA, 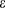 is less than 1.0. 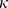 varies in the range of 2.0–5.0. The parameters 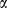 and 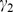 are experimentally controlled and vary from 0–0.4 and 0–2.5, respectively, and 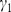 is set as 1.0 [31], [34].

**Figure 3:**
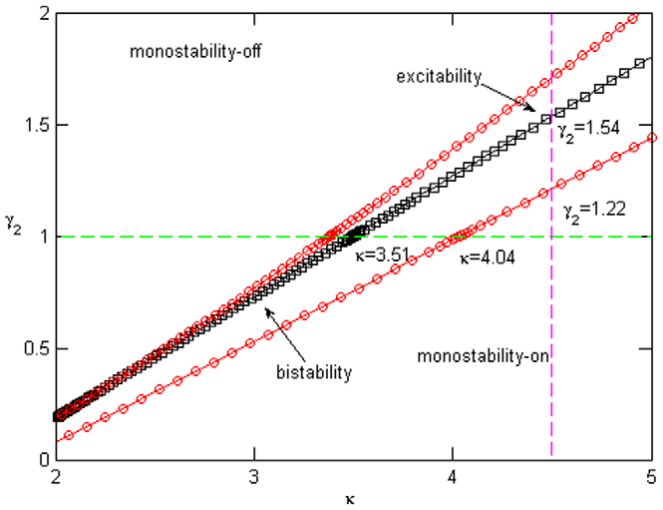
The bifurcation diagram spanned by the positive feedback (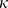) and the miRNAs inhibition (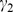). The red circles and black squares on borderlines represent saddle-nodes and Hopf bifurcations, respectively. The diagram includes three features: monostability, bistability, and excitability. The green and plum dashed lines denote the cases in which 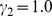 and 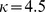, respectively. The parameter values are 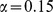, 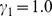, and 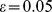.

The protein and miRNAs in the interlinked Myc/E2F/miR-17-92 network have been shown to transition from monostability to excitability to bistability, then to monostability with increasing 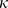 [31], [34]. A practical way of producing diverse signal-response behaviors is to combine 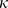, the positive feedback loop, with 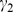, the inhibition of miRNAs [34]. Figure 3 presents an overview of the tunable dynamics in the 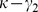 plane.

**Figure 4:**
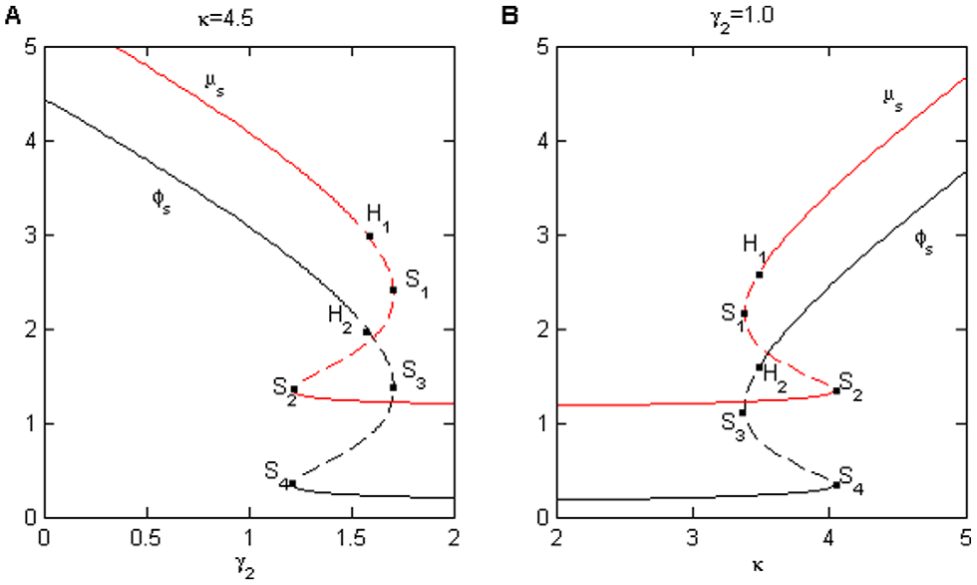
The steady-state bifurcation diagrams of the protein concentration 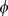 (black line) and miRNA concentration 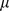 (red line) for (A) 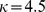 and (B) 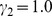 with increasing 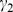 and 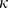, respectively. 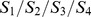
 denote saddle points and 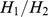 represent a Hopf bifurcations. Clearly, the system has completed the transitions (A) from monostability to bistability to excitability, and has finally transitioned to monostability with increasing 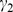 or (B) from monostability to excitability to bistability, and has finally transitioned to monostability with increasing 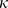. The parameter values are 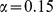, 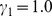, 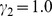, and 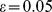.

Three types of dynamics exist: monostability, bistability, and excitability. Note that we denote the low protein steady state as the *off* state and the high protein steady state as the *on* state. In monostability, a system remains in a stable state (on/off), when it is perturbed. A bistable system exhibits an unstable state and two stable states [35]. During excitability, there is a rest state, an excited state, and a recovery state. The system resides in the rest state if it is unperturbed. However, the system can leave the rest state in response to a sufficiently strong input stimulus, undergoing firing and refractory states before it returns to a rest state [36]. As shown in Figure 4B, the region from saddle node 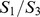 to Hopf bifurcation node 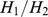 represents excitability (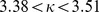), the region from 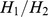 to saddle node bifurcation 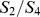 represents the bistability (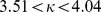), and the other regions are monostability. When 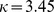 (excitability), there is only one stable state. With a pulse from 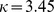 to 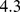 (monostability-on state), the system exhibits excitability by first coming into a stable state, then jumping into a higher stable state for 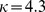, and finally dropping into the previous stable state post-pulse. However, when the system appears bistable (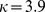), as in the case of a pulse from 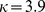 to 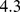, it undergoes a trajectory from the lower stable state to the higher state for 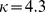, and then to a higher stable state for 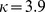.

In this work, considering that the contribution of extrinsic noise is dominant [2], we assume that the protein input signal fluctuates around its mean value 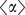, 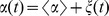. 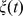 denotes the extrinsic noise fluctuation. Since colored noise is more realistic, especially in biological systems [37], the autocorrelation time of 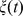 is non-negligible and is related to the cell cycle [9], [38], [39]. Thus, the noise involved in this interlinked loop is described by
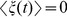
(5)

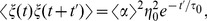
(6)where 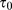 is the autocorrelation time of the noise and 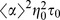 is the noise intensity. In other words, 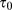 describes the noise bandwidth and 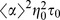 represents the spectral height of the colored noise [40].

### Signal sensitivity and noise amplification

To study the propagation of an input signal with noise, we define the steady-state sensitivity susceptibility 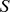 as a measure of the change in the output signal due to a change in the input signal [41]–[43],
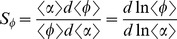
(7)

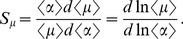
(8)Here, 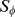 and 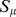 represent the relative change in protein and miRNAs in response to changes in the input signal, respectively. The symbol 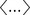 means that the parameters are measured at the stable state.

To obtain the noise propagation from the input signal to the output signal, we measure the noise amplification 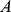, which is defined as the ratio between the output noise and the input noise [25],
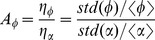
(9)

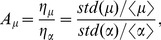
(10)where 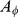 and 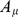 denote the noise amplification of protein and miRNAs due to fluctuations in the input signal, respectively. The variables 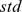 and 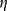 represent the standard deviation and the relative standard deviation, respectively. Clearly, for 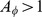 (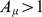), the noise of 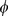 (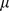) is propagated and amplified, whereas the noise of 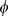 (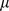) is repressed when 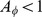 (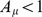).

The detailed deduction process to calculate the signal sensitivity and noise amplification formulas (Eq. (3–4)) is presented in Text S1.

### Interlinked positive and negative feedback loop

As stated above, the system has a transition from monostability to excitability, then switches to bistability before it finally reaches monostability with an increased intensity of negative feedback 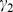. Switching behaviors from the bistability are commonly reported. These behaviors are important in the biological processes, such as circadian clocks [44], [45], the eukaryotic cell cycle [46], [47], the p53-Mdm2 network [48], and so on. Therefore, in this work, we mainly focus on the gene network's bistable behaviors that involve interlinked positive and negative feedback loops. Note that in bistability, the system's stable state is determined by its initial state. As mentioned above, the higher and the lower steady states are normally referred to as the on-state and the off-state, respectively. Thus, in the following section, we study the bistability region in two different cases, one in which the initial steady state is in the on-state and the other when it is in the off-state.

**Figure 5:**
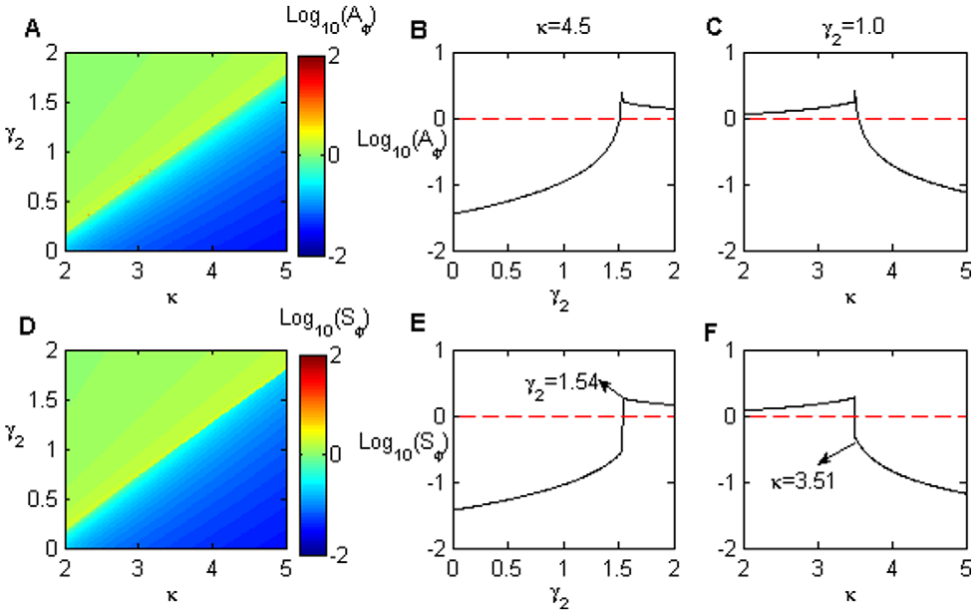
The effects of the positive feedback (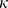) and miRNA inhibition (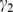) with initial steady on-state. (A) The noise amplification and (D) the sensitivity of the protein module as a function of 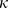 and 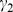 when the initial stable steady state is on-state in a bistable region. 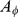 and 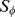 for 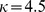 (B, E) and 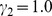 (C, F), respectively. Note that 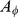 and 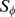 reach their maximum values at 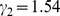 (B, E) and 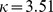 (C, F), respectively. The parameters values are 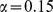, 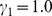, 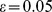, and 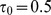.

For an initial stable steady state in the on-state, Figure 5A presents a bulk diagram of the noise amplification 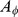 as a function of the positive feedback loop 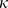 and the negative feedback loop 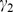. The diagram can be divided into two parts based on the Hopf bifurcation line, which is marked with a black square in Figure 3. The upper, warmer-colored pattern in Figure 5A corresponds to the monostability and excitability regions, and the lower, cooler-colored pattern corresponds to the on-state in the bistability and monostability regions (also see Figure 3). In the upper region, 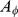 is larger than 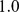, and it increases as 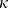 increases, but decreases as 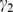 increases.In the lower region, 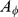 is less than 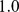 and it decreases as 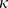 increases, but 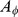 is amplified as 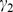 increases.

Figure 5B illustrates the noise amplification 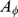 for 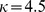. Clearly, a maximal 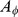 exists at 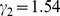 in Figure 5B. This value corresponds to the Hopf bifurcation 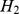 in the steady-state bifurcation diagrams of the protein concentration 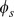 (see Figure 3 and 4A). In the region where 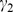 is less than its value at the maximal point, 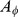 is always less than 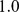, even though it increases with increasing 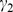. Note that this parameter region corresponds to the on-state of the systems (the lower area in Figure 5A). Therefore, the noise is buffered in the on-state, but the repression ability is reduced when approaching the Hopf bifurcation value. Similarly, when 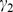 is larger than its value at the maximal point (the upper region of Figure 5A), the system is in the off-state. The noise is amplified when it comes into the excitability region (because 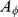 always is larger 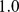), and and this amplifying ability is enhanced as the value approaches the Hopf bifurcation.

Figure 5C plots the 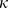 dependence of 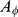 in an example with miRNA inhibition of 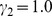. When the system is initially in the off-state, the noise is clearly amplified (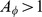) in the monostability and excitability regions. This amplification ability is improved by increasing 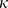 until it reaches its maximum value at 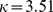. However, after crossing this maximum point (the Hopf bifurcation point 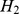 in Figure 4B), the system transitions into an on-state, 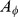 quickly reduces to less than 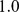 and 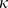 begins to repress the noise.

Figures 5D–F present the signal sensitivity 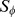 in terms of 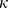 and 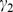. The dependence properties of 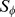 are compared to those of 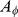. Similar to Figures 5A, the bulk diagram of 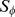 is also divided into two regions by the Hopf bifurcation points from Figure 3. In the upper region (the off-state), 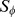 increases with increasing 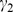 or decreasing 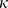, whereas it decreases with decreasing 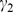 or increasing 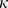, in the lower region (the on-state).

**Figure 6 pone-0051840-g006:**
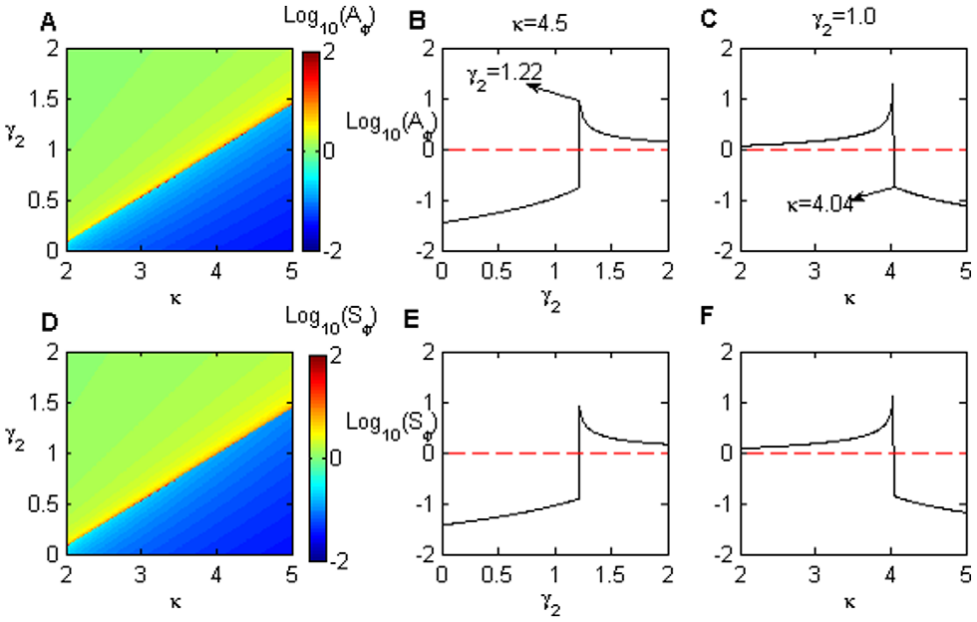
The effects of the positive feedback (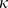) and miRNA inhibition (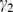) with an initial steady off-state. (A) The noise amplification and (D) the sensitivity of the protein module as a function of 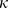 and 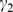 when the initial steady state is an off-state in the bistable region. The parameter values are 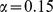, 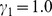, 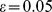, 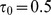. 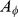 and 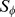 for 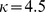 (B, E) and 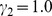 (C, F), respectively. Note that 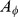 and 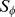 reach their maximum values at 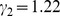 (B, E) and 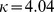 (C, F), respectively.

For the case in which the initial stable steady is the off-state, we plot the bulk diagrams of 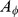 and 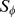 as the function with 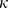 and 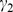 in Figure 6. It is clear that the 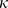 and 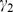 dependence of 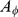/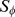 is the same as the case described in Figure 5. The only difference between Figures 5 and 6 is that the boundary line in Figures 6A and 6D represent the saddle nodes (marked with red circles in Figure 3) rather than the Hopf bifurcation points in Figures 5A and 5D. The maximal 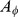 and 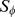 values are located on the lower saddle-node boundary of the bistability region in Figure 3. The upper regions of Figures 6A and 6D correspondto the off-states, including monostability, excitability, and bistability. However lower regions only represent the on-state in monostability.

Clearly, because the deviation between the steady states of the miRNA and protein modules is constant [31], [34], the noise amplification 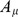 and the signal sensitivity 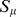 of the miRNA should have the same evolutionary tendencies. In fact, we observe similar 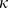/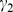-dependent behaviors in 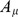 and 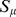, but 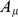 is always less than 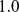 based on the dynamic properties of the system (see Figures 1 and 2 in Text S2).

These results indicate that the role of interlinked positive and negative feedback loops is not a simple noise suppressor or a noise amplifier. The effects of positive and negative feedback loops are dependent on the dynamic properties and initial state of the system. In the case of an initial on-state, noise propagation is always suppressed, but the the miRNA's negative feedback loop enhances the signal sensitivity and noise amplification. When there is an initial off-state, the negative feedback loop begins to reduce the noise amplification and signal sensitivity. The positive feedback loop is always contrary to the negative feedback loop in all cases. It is clear that the role of interlinked feedback loops appears to be a noise buffer, based on both of its components. Moreover, the boundary line between the two different roles is determined by the bistability properties of the system (see Figures 5, 6).

### Autocorrelation time of the input noise and time scale of the reactions

It is well known from previous experiments that the extrinsic noise is usually colored noise with a long autocorrelation time [38], [49], [50]. When the noise autocorrelation time 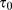 is lower than the relaxation time of the system, the noise should not be neglected. 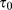 is also crucial to system behavior. Hornung et al. reported that noise amplification is buffered by decreasing noise autocorrelation times in linear networks [25].

In terms of cellular regulation, gene transcription networks can be considered a slow reaction when the time scale of the network is minutes, and reactions with a sub-second timescale are considered fast reactions [51]. In systems with a single-loop switch, a fast-loop switch can induce rapid responses. miRNA has been found to diversify the response behaviors of a system based on its input stimulus [34], [52]. The research on budding yeast polarization shows that slow positive feedback loop is in favor of the stability of the polarized on-state [53]. In the Ca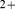 signaling, the long-term Ca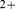 signals are robust in response to weak stimuli and noise [54].

As presented above, the properties of noise amplification and signal sensitivity in a system with interlinked feedback are dependent on its dynamic status and its initial state. However, we observed that the autocorrelation time 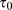 and the time scale of the protein reaction 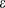 did not have an effect on the signal sensitivity 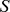 (see Figures 1–4 in Text S3).

**Figure 7:**
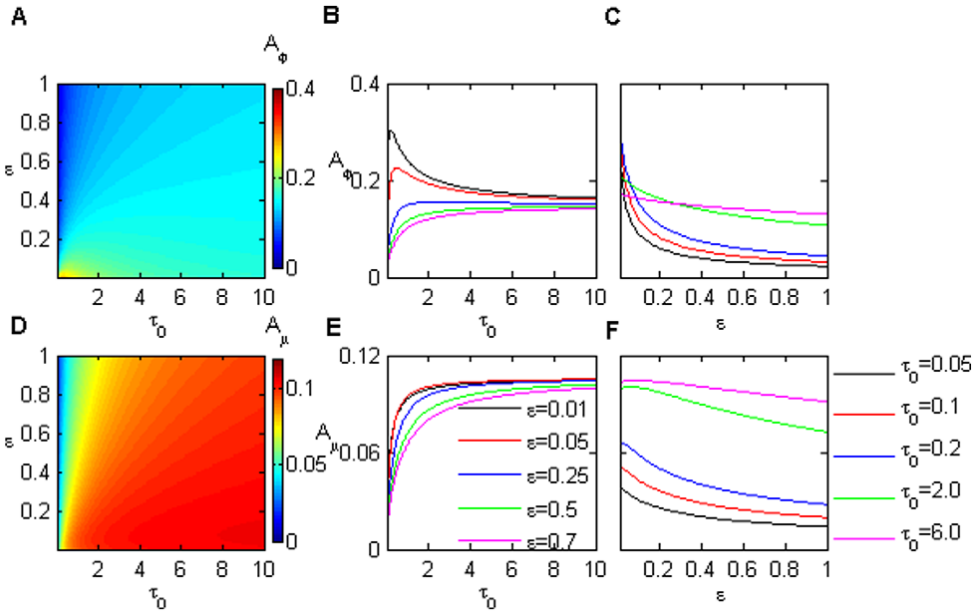
The role of the noise autocorrelation time 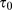 and the time scale of the protein reaction 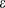 on noise amplification. The noise amplification in (A) the protein module and (D) miRNAs as a function of 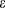 and 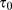. The noise amplification evolutes with 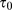 in input signal for various 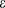 (B, E), and 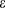 for different 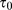 (C, F) for protein and miRNA modules, respectively. The parameter values are 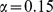, 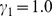, 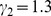, 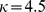.

The noise amplification 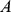 is influenced by 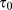 and 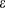, especially in the region of lower parameter values. Figures 7A and 7D illustrate 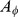 and 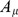 as the function of 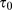 and 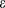, respectively, in the case of an initial steady on-state in the bistable region (similar behaviors were observed in other cases; see Figures 5–7 in Text S3). Both noise amplifications (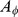/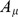) increase quickly and then tend towards their saturation values with increasing 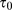. The noise saturation values decrease with increasing 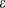. However, when 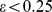, 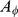 sharply increases at first, then decreases with increasing 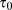. This behavior indicates that a maximal noise amplification value possibly exists with a smaller 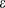 (faster reaction) and a smaller 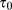 (shorter noise autocorrelation time). In addition, in the same parameter region, 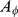 declines faster than 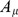 with increasing 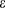.

For example, in Figure 7B, 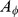 first increases to a maximum, then it reduces to a stationary value for smaller 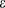 as 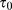 increases. When 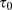 is slightly larger, 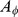 increases directly to its saturation value, similar to 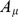 in Figure 7E. In the case of a fixed 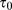 (Figures 7C and 7F), both 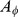 and 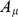 reduce with increasing 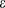. The smaller 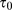, the more 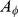 and 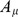 quickly decrease. Moreover, 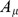 decreases more slowly than 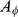 because miRNA is more stable than protein.

Therefore, decreasing the autocorrelation time of the noise and increasing the time scales of the reaction can reduce noise amplification without compromising signal sensitivity. Increasing the timescale of the extrinsic fluctuations can enhance a noise effect in the feedforward loops [39]. Slow feedback loops are critical for some cellular processes. For example, in the Xenopus oocyte's maturation period, interkinesis occurred with Cdc2 partially deactivated [55]. The slow positive feedback loop between Cdc2 and the MAPK cascade can maintain an on-state rather than transition to an off-state [52]. If the bistable system stay at off-state, like the transcriptional factor E2F, as the level of E2F is off-state without proper serum [56], however, a rapid feedback loop can induce E2F to remain in the on-state to control the cell cycle [57]. While, overexpression E2F-1 can promote neoplastic transformation [58]. Therefore, complex circuits can be properly constructed in terms of fitness.

## Discussion

It has been reported that interlinked positive and negative feedback loops can act as tunable modules for superior adaptability and robustness [32]. In previous studies, it was reported that a single positive feedback loops can buffer the noise [25], while a single negative feedback loop can optimize noise repression levels within a defined range [59]. A role for coupled positive and negative feedback motifs has been reported in cellular signaling pathways, such as the PDGF signaling pathway [60], muscle cell fate specification [61], [62], Ca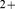 signaling [63], and so on. In addition, mathematical equations have been created [64], [65] to observe the specific regulatory functions of coupled feedback loops in cellular circuits. Positive feedback loops have been shown to turn on in response to a stimulus and robustly remain in the on-state, whereas negative feedback loops can suppress a sustained response. These observations are in accordance with previous experimental results [60]–[62]. In Ref. [66], it was shown that the higher concentration of the positice feedback can induce the off-cells switch to the on-state in a stochastic way. It was also theoretically investigated that the negative feedback reduces the variance of the noise in the higher protein level [67]. Thus, interlinked positive and negative feedback loops can properly modulate signal responses and effectively handle noise, enabling them to make reliable decisions in signaling pathways.

The response of interlinked positive and negative feedback loops to stochastic fluctuations has not been thoroughly examined in a bistable system with various dynamic properties. Feedback loops play a critical role in noise propagation. The E2F/Myc/miR-17-92 network has various dynamic properties (e.g., monostability, bistability, excitability; see Figure 3) that are fine-tuned by positive and negative feedback loops. Thus, the tunable effect of interlinked feedback loops on signal sensitivity and noise propagation is expected to be much different than that of single positive/negative feedback loops. The present work shows that interlinked positive and negative feedback loops dynamically tune noise propagation signals rather than monotonically suppressing or amplifying these signals, as would be expected in single feedback loops.

In this paper, we used frequency domain analysis [25], [67], [68] to obtain expressions of systemic noise amplification and signal sensitivity and to show that the roles of the positive and negative feedback loops are dependent on dynamic behaviors. This deduced recipe is consistent with the stochastic simulation result from Gillespie algorithm with an acceptable deviation (See Text S4). Noise amplification and signal sensitivity behaviors change in various regions depending on the levels of positive and negative feedback.

The positive/negative feedback in an interlinked feedback loop does not simply suppress or enhance noise. For example, Figures 5 and 6 show that the miRNA negative feedback loop reduces noise buffering to improve the signal sensitivity. This buffering is achieved by slightly compromising on noise robustness when the noise amplification (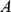) is less than 1. When 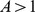, the negative feedback loop appears to suppress noise. In addition to these synergetic, tunable behaviors between the positive and negative feedback loops, it is interesting to note that the noise autocorrelation time 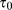 and the response time constant of the protein module 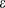 have no effect on the signal sensitivity (Figures 1–4 in Text S3). Thus, the noise amplification can be tuned to a fixed signal sensitivity based on the effects of 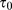 and 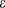 on 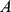 (see Figure 7 and Figures 5–7 in Text S3).

In addition, due to the underlying biochemical reactions, gene regulatory networks involve time delays. Thus, the E2F/Myc/miR-17-92 network should also consider the effects of time delays. It is well known that long time delays can generate oscillations in a negative feedback loop, and that the oscillations amplitude can be increased by the addition of positive feedback loop [32], [69], [70]. Therefore, we assume that there will not be any qualitative changes in our results by including time delays in our systemic signal sensitivity and noise amplification.

## Materials and Methods

Bifurcation analyses of the ordinary differential equations are performed with OSCILL 8.28 [71]. The integral is calculated in Mathematica 7.01.0 for Linux. The ordinary differential equations were numerically solved using the fourth-order Runge-Kutta scheme [72] in Fortran 95 codes.

## Supporting Information

Text S1
**The deduction processes for the signal sensitivity** 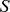 **and the noise amplification** 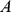 **.**
(PDF)

Text S2
**Effects of the positive feedback loop** 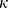 **and miRNA inhibition** 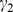 **on noise amplification and the sensitivity of the miRNA module when the initial steady is in the on-state or the off-state in a bistable region.**
(PDF)

Text S3
**Effects of the noise autocorrelation time** 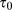 **and the time scale of the protein reaction** 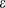 **on signal sensitivity and noise amplification for the system with an initial steady in the on-state or the off-state in a bistable region and an on/off state in a monostable region.**
(PDF)

Text S4 **The noise amplification from the stochastic simulation results with Gillespie algorithm and from the deduced results Equation (42) in Text S1.**
(PDF)
