# Supplementary material for: Noise Propagation in Gene Regulation Networks Involving Interlinked Positive and Negative Feedback Loops
Source: PLoS One. 2012 Dec 20;7(12):e51840. doi: 10.1371/journal.pone.0051840 (PMC3527455; doi:10.1371/journal.pone.0051840)
Supplement: Text S1 — The deduction processes for the signal sensitivity and the noise amplification . (PDF) [file pone.0051840.s001.pdf]

Noise propagation in gene regulation networks involving interlinked positive and negative feedback loops (*Supplementary Text S1*)

Hui Zhang, Yueling Chen, Yong Chen*

* E-mail: ychen@lzu.edu.cn

1 Signal sensitivity and noise amplification

To study the propagation of the input signal involving noise, we define the steady-state sensitivity susceptibility S to measure the change in the output signal due to the input change [1–3],

$$S_\phi = \frac{\langle\alpha\rangle d\langle\phi\rangle}{\langle\phi\rangle d\langle\alpha\rangle} = \frac{dln\langle\phi\rangle}{dln\langle\alpha\rangle} \quad (1)$$

$$S_\mu = \frac{\langle\alpha\rangle d\langle\mu\rangle}{\langle\mu\rangle d\langle\alpha\rangle} = \frac{dln\langle\mu\rangle}{dln\langle\alpha\rangle}. \quad (2)$$

Here S_ϕ and S_μ represent the relative change of protein and miRNAs following the change in the input signal, respectively. $\langle ... \rangle$ means the parameters are measured at the stable state.

The abstract dynamics of the protein-miRNAs network is the following [4, 5]

$$\varepsilon\frac{d\phi}{dt} = \alpha + \left(\frac{\kappa\phi^2}{\gamma_1 + \phi^2 + \gamma_2\mu}\right) - \phi \quad (3)$$

$$\frac{d\mu}{dt} = 1 + \phi - \mu. \quad (4)$$

To briefly describe, Eqs. (3-4) are explicitly rewritten by

$$\frac{d\phi}{dt} = J_1^+(\alpha,\phi,\mu) - J_1^-(\alpha,\phi,\mu) \quad (5)$$

$$\frac{d\mu}{dt} = J_2^+(\phi,\mu) - J_2^-(\phi,\mu), \quad (6)$$

where J_1^+ and J_1^- (J_2^+ and J_2^-) are the total fluxes of production and elimination of protein (miRNAs), respectively. Comparing with Eqs. (3) and (4), we have

$$J_1^+ = \frac{1}{\varepsilon}\left(\alpha + \left(\frac{\kappa\phi^2}{\gamma_1 + \phi^2 + \gamma_2\mu}\right)\right) \quad (7)$$

$$J_1^- = \frac{1}{\varepsilon}\phi \quad (8)$$

$$J_2^+ = 1 + \phi \quad (9)$$

$$J_2^- = \mu. \quad (10)$$

When the system locates in a stable steady state, the protein and miRNAs are not changed with time. So Eqs. (5) and (6) are equal to zero. It means

$$\langle J_1^+\rangle = \langle J_1^-\rangle = \langle J_1\rangle \quad (11)$$

$$\langle J_2^+\rangle = \langle J_2^-\rangle = \langle J_2\rangle. \quad (12)$$

Based on frequency domain analysis [6–8], we differentiate Eqs. (5) and (6) with respect to α at the steady state by using the chain rule, and then multiply with $\langle\alpha\rangle/\langle J_1\rangle$ and $\langle\alpha\rangle/\langle J_2\rangle$, respectively,

$$\frac{\langle\alpha\rangle}{\langle J_1\rangle}\left[\frac{\partial\left(\langle J_1^+\rangle - \langle J_1^-\rangle\right)}{\partial\langle\alpha\rangle} + \frac{\partial\left(\langle J_1^+\rangle - \langle J_1^-\rangle\right)}{\partial\langle\phi\rangle}\frac{d\langle\phi\rangle}{d\langle\alpha\rangle} + \frac{\partial\left(\langle J_1^+\rangle - \langle J_1^-\rangle\right)}{\partial\langle\mu\rangle}\frac{d\langle\mu\rangle}{d\langle\alpha\rangle}\right] = 0 \quad (13)$$

$$\frac{\langle\alpha\rangle}{\langle J_2\rangle}\left[\frac{\partial\left(\langle J_2^+\rangle - \langle J_2^-\rangle\right)}{\partial\langle\alpha\rangle} + \frac{\partial\left(\langle J_2^+\rangle - \langle J_2^-\rangle\right)}{\partial\langle\phi\rangle}\frac{d\langle\phi\rangle}{d\langle\alpha\rangle} + \frac{\partial\left(\langle J_2^+\rangle - \langle J_2^-\rangle\right)}{\partial\langle\mu\rangle}\frac{d\langle\mu\rangle}{d\langle\alpha\rangle}\right] = 0. \quad (14)$$

Considering the definition of the reaction flux elasticites H_{ij} [1,2], it should be

$$H_{ij} = -\frac{\langle n_i \rangle}{\langle J_j \rangle}\left(\frac{\partial \langle J_i^+ \rangle}{\partial \langle n_j \rangle} - \frac{\partial \langle J_i^- \rangle}{\partial \langle n_j \rangle}\right) = -\frac{\langle n_j \rangle}{\langle J_j \rangle} A_{ij}, \tag{15}$$

where we use n_0, n_1, n_2 in substitution of α, ϕ, μ, and $A_{ij} = \frac{\partial}{\partial \langle n_j \rangle}\frac{\partial \langle n_i \rangle}{\partial t}$ with $i = 1$, 2 and $j = 0$, 1, 2.

Finally, Eqs. (13) and (14) are rewritten by

$$H_{10} + H_{11}S_1 + H_{12}S_2 = 0 \tag{16}$$

$$H_{20} + H_{21}S_1 + H_{22}S_2 = 0, \tag{17}$$

where S_ϕ and S_μ are replaced by S_1 and S_2 to represent the susceptibility of the protein and the miRNAs, respectively. H_{ij} are

$$H_{10} = -\frac{\langle \alpha \rangle}{\langle \phi \rangle} \tag{18}$$

$$H_{11} = 1 - \frac{2\kappa \langle \phi \rangle (\gamma_1 + \gamma_2 \langle \mu \rangle)}{(\gamma_1 + \langle \phi \rangle^2 + \gamma_2 \langle \mu \rangle)^2} \tag{19}$$

$$H_{12} = -\frac{\gamma_2 \kappa \langle \phi \rangle \langle \mu \rangle}{(\gamma_1 + \langle \phi \rangle^2 + \gamma_2 \langle \mu \rangle)^2} \tag{20}$$

$$H_{20} = 0 \tag{21}$$

$$H_{21} = -\frac{\langle \phi \rangle}{\langle \mu \rangle} \tag{22}$$

$$H_{22} = 1. \tag{23}$$

Here, note that all quantities are measured at the steady state.

From Eqs. (16) and (17), the noise susceptibilities of the protein and miRNAs are,

$$S_\phi = S_1 = \left|\frac{H_{20}H_{12} - H_{10}H_{22}}{H_{11}H_{22} - H_{21}H_{12}}\right| \tag{24}$$

$$S_\mu = S_2 = \left|\frac{H_{10}H_{21} - H_{11}H_{20}}{H_{11}H_{22} - H_{12}H_{21}}\right|. \tag{25}$$

Here, $S_\phi < 1$ ($S_\mu < 1$) means the signal sensitivity is relatively low or the changes of protein (miRNAs) module is less than that of input signal. On the contrary, $S_\phi > 1$ ($S_\mu > 1$) means a higher signal sensitivity and the changes of protein (miRNAs) module is larger than that of input signal. Clearly, a lager value of S_ϕ and S_μ is of great benefit to biological networks.

To obtain the noise propagation from the input signal to the output, there is another measurement, the noise amplification A, that is defined as the ratio between the output noise and input [6]

$$A_\phi = \frac{\eta_\phi}{\eta_\alpha} = \frac{std(\phi)/\langle \phi \rangle}{std(\alpha)/\langle \alpha \rangle} \tag{26}$$

$$A_\mu = \frac{\eta_\mu}{\eta_\alpha} = \frac{std(\mu)/\langle \mu \rangle}{std(\alpha)/\langle \alpha \rangle}, \tag{27}$$

where A_ϕ and A_μ denote the noise amplification of protein and miRNAs due to the fluctuation in the input signal, respectively. std and η represent the standard deviation and the relative standard deviation, respectively. Clearly, for $A_\phi > 1$ ($A_\mu > 1$), the noise involved in ϕ (μ) is propagated and amplified, vice versa.

Similarly, using the approach of frequency domain analysis [7,9], in terms of steady state fluctuations $\Delta n_0 = n_0(t) - \langle n_0 \rangle$, $\Delta n_1 = n_1(t) - \langle n_1 \rangle$, $\Delta n_2 = n_2(t) - \langle n_2 \rangle$, we rewrite Eqs. (5) and (6) with

$$\frac{d\Delta n_1(t)}{dt} = \frac{\partial(\langle J_1^+ \rangle - \langle J_1^- \rangle)}{\partial \langle n_0 \rangle}\Delta n_0 + \frac{\partial(\langle J_1^+ \rangle - \langle J_1^- \rangle)}{\partial \langle n_1 \rangle}\Delta n_1 + \frac{\partial(\langle J_1^+ \rangle - \langle J_1^- \rangle)}{\partial \langle n_2 \rangle}\Delta n_2 \tag{28}$$

$$\frac{d\Delta n_2(t)}{dt} = \frac{\partial(\langle J_2^+ \rangle - \langle J_2^- \rangle)}{\partial \langle n_0 \rangle}\Delta n_0 + \frac{\partial(\langle J_2^+ \rangle - \langle J_2^- \rangle)}{\partial \langle n_1 \rangle}\Delta n_1 + \frac{\partial(\langle J_2^+ \rangle - \langle J_2^- \rangle)}{\partial \langle n_2 \rangle}\Delta n_2. \tag{29}$$

And then dividing the above equations by J_1 and J_2 respectively, one obtains

$$\frac{\langle n_1\rangle}{\langle J_1\rangle}\frac{dx_1}{dt} = \frac{\langle n_0\rangle}{\langle J_1\rangle}\frac{\partial(\langle J_1^+\rangle - \langle J_1^-\rangle)}{\partial\langle n_0\rangle}x_0 + \frac{\langle n_1\rangle}{\langle J_1\rangle}\frac{\partial(\langle J_1^+\rangle - \langle J_1^-\rangle)}{\partial\langle n_1\rangle}x_1 + \frac{\langle n_2\rangle}{\langle J_1\rangle}\frac{\partial(\langle J_1^+\rangle - \langle J_1^-\rangle)}{\partial\langle n_2\rangle}x_2 \quad (30)$$

$$\frac{\langle n_2\rangle}{\langle J_2\rangle}\frac{dx_2}{dt} = \frac{\langle n_0\rangle}{\langle J_2\rangle}\frac{\partial(\langle J_2^+\rangle - \langle J_2^-\rangle)}{\partial\langle n_0\rangle}x_0 + \frac{\langle n_1\rangle}{\langle J_2\rangle}\frac{\partial(\langle J_2^+\rangle - \langle J_2^-\rangle)}{\partial\langle n_1\rangle}x_1 + \frac{\langle n_2\rangle}{\langle J_2\rangle}\frac{\partial(\langle J_2^+\rangle - \langle J_2^-\rangle)}{\partial\langle n_2\rangle}x_2, \quad (31)$$

where $x_0(t) = (n_0(t) - \langle n_0\rangle)/\langle n_0\rangle$, $x_1(t) = (n_1(t) - \langle n_1\rangle)/\langle n_1\rangle$, and $x_2(t) = (n_2(t) - \langle n_2\rangle)/\langle n_2\rangle$. According to Eqs. (14-23), we rewrite the above equations with

$$-\tau_1\frac{dx_1}{dt} = H_{10}x_0 + H_{11}x_1 + H_{12}x_2 \quad (32)$$

$$-\tau_2\frac{dx_2}{dt} = H_{20}x_0 + H_{21}x_1 + H_{22}x_2, \quad (33)$$

where $\tau_1 = \langle n_1\rangle/J_1$ and $\tau_2 = \langle n_2\rangle/J_2$.

To transform the time domain to the frequency domain, we perform the Fourier transformations on Eqs. (32) and (33), $\hat{x_1}(\omega) = \int_{-\infty}^{\infty} x_1(t)e^{-i\omega t}dt$ and $\hat{x_2}(\omega) = \int_{-\infty}^{\infty} x_2(t)e^{-i\omega t}dt$. One obtains the following relations,

$$\hat{x_1}(\omega) = \frac{H_{10}(H_{22} + i\omega\tau_2) - H_{20}H_{12}}{H_{21}H_{12} - (H_{11} + i\omega\tau_1)(H_{22} + i\omega\tau_2)}\hat{x_0}(\omega) \quad (34)$$

$$\hat{x_2}(\omega) = \frac{H_{20}(H_{11} + i\omega\tau_1) - H_{10}H_{21}}{H_{21}H_{12} - (H_{11} + i\omega\tau_1)(H_{22} + i\omega\tau_2)}\hat{x_0}(\omega). \quad (35)$$

Here, $\hat{x_0}(\omega)$ presents a connection between the frequency response x_1 and x_2, and that of the input signal.

Due to the input fluctuation: $\langle \xi(t)\xi(t+t')\rangle = \langle\alpha\rangle^2\eta_0^2 e^{-t'/\tau_0}$, we get the correlation function of $x_0(t)$, with magnitude η_0^2

$$\langle x_0(t)x_0(t+t')\rangle = \eta_0^2 e^{-t'/\tau_0}. \quad (36)$$

Therefore, the Fourier transformation for Eq. (36) is

$$\langle \hat{x_0}(\omega)\hat{x_0}^*(\omega)\rangle = 2\eta_0^2\frac{\tau_0}{\tau_0^2\omega^2 + 1}, \quad (37)$$

where $\hat{x_0}^*(\omega)$ denotes the complex conjugate. Combining it with Eq. (34) and (35), we obtain the power spectrums of x_1 and x_2,

$$\langle \hat{x_1}(\omega)\hat{x_1}^*(\omega)\rangle = \frac{(H_{10}H_{22} - H_{20}H_{12})^2 + \omega^2 H_{10}^2\tau_2^2}{(H_{21}H_{12} - H_{11}H_{22} + \omega^2\tau_1\tau_2)^2 + \omega^2(\tau_1 H_{22} + \tau_2 H_{11})^2}\langle \hat{x_0}(\omega)\hat{x_0}^*(\omega)\rangle \quad (38)$$

$$\langle \hat{x_2}(\omega)\hat{x_2}^*(\omega)\rangle = \frac{(H_{20}H_{11} - H_{10}H_{21})^2 + \omega^2 H_{20}^2\tau_1^2}{(H_{21}H_{12} - H_{11}H_{22} + \omega^2\tau_1\tau_2)^2 + \omega^2(\tau_1 H_{22} + \tau_2 H_{11})^2}\langle \hat{x_0}(\omega)\hat{x_0}^*(\omega)\rangle. \quad (39)$$

Now, in order to obtain the noise magnitude η_1 and η_2, we transform the above equations back to the time domain and replace $t' = 0$ in the autocorrelation function,

$$\eta_1^2 = \frac{1}{2\pi}\int_{-\infty}^{\infty}\langle \hat{x_1}(\omega)\hat{x_1}^*(\omega)\rangle e^{i\omega t'}d\omega\big|_{t'=0} = \frac{1}{2\pi}\int_{-\infty}^{\infty}\langle \hat{x_1}(\omega)\hat{x_1}^*(\omega)\rangle d\omega \quad (40)$$

$$\eta_2^2 = \frac{1}{2\pi}\int_{-\infty}^{\infty}\langle \hat{x_2}(\omega)\hat{x_2}^*(\omega)\rangle e^{i\omega t'}d\omega\big|_{t'=0} = \frac{1}{2\pi}\int_{-\infty}^{\infty}\langle \hat{x_2}(\omega)\hat{x_2}^*(\omega)\rangle d\omega. \quad (41)$$

Substituting Eqs. (40-41) into Eqs. (26-27), we get the noise amplifications of A_ϕ and A_μ,

$$
\begin{aligned}
A_\phi &= \left[i\tau_0 \left(\frac{\sqrt{2}[2(H_{10}H_{22}\tau_1\tau_2)^2 - bH_{10}^2]}{c(\tau_1 H_{22} + \tau_2 H_{11})\sqrt{-b}/\tau_1\tau_2} + \frac{\sqrt{2}[-2(H_{10}H_{22}\tau_1\tau_2)^2 + dH_{10}^2]}{e(\tau_1 H_{22} + \tau_2 H_{11})e\sqrt{-d}\tau_1\tau_2} \right) \right. \\
&+ \left. \frac{\tau_0^2[-H_{10}^2 + (H_{10}H_{22})^2\tau_0^2]}{(\tau_1\tau_2)^2 - [b + a(\tau_1 H_{22} + \tau_2 H_{11})]\tau_0^2 + (H_{21}H_{12} - H_{11}H_{22})^2\tau_0^4} \right]^{1/2} \\
A_\mu &= \left[i\tau_0 \left(\frac{2\sqrt{2}(-H_{10}H_{21}\tau_1\tau_2)^2}{c(\tau_1 H_{22} + \tau_2 H_{11})\sqrt{-b}/\tau_1\tau_2} + \frac{-2\sqrt{2}(-H_{10}H_{21}\tau_1\tau_2)^2}{e(\tau_1 H_{22} + \tau_2 H_{11})e\sqrt{-d}\tau_1\tau_2} \right) \right. \\
&+ \left. \frac{(H_{20}H_{11} - H_{10}H_{21})^2\tau_0^4}{(\tau_1\tau_2)^2 - [b + a(\tau_1 H_{22} + \tau_2 H_{11})]\tau_0^2 + (H_{21}H_{12} - H_{11}H_{22})^2\tau_0^4} \right]^{1/2}, \qquad (42)
\end{aligned}
$$

where the parameters are

$$
\begin{aligned}
a &= \sqrt{4(H_{21}H_{12} - H_{11}H_{22})\tau_2 + (\tau_1 H_{22} + \tau_2 H_{11})^2} \\
b &= 2(H_{21}H_{12} - H_{11}H_{22})\tau_1\tau_2 + (\tau_1 H_{22} + \tau_2 H_{11})^2 - a(\tau_1 H_{22} + \tau_2 H_{11}) \\
c &= a[2(\tau_1\tau_2)^2 - b\tau_0^2] \\
d &= 2(H_{21}H_{12} - H_{11}H_{22})\tau_1\tau_2 + (\tau_1 H_{22} + \tau_2 H_{11})^2 + a(\tau_1 H_{22} + \tau_2 H_{11}) \\
e &= a[2(\tau_1\tau_2)^2 - d\tau_0^2].
\end{aligned}
$$

Obviously, a smaller value of A_ϕ (A_μ) has benefit of repressing the noise propagation in biological networks. Especially $A < 1$ means that the signal fluctuation should decline in propagation on the networks. In contrast, for $A_\phi > 1$ ($A_\mu > 1$), the fluctuations in output is larger than that in the input signal, or the noise is amplified in the network.

References

1. Paulsson J (2004) Summing up the noise in gene networks. Nature 427: 415-418.

2. Paulsson J (2005) Models of stochastic gene expression. Phys Life Rev 2: 157-175.

3. Swain PS, Elowitz MB, Siggia ED (2002) Intrinsic and extrinsic contributions to stochasticity in gene expression. Proc Natl Acad Sci U S A 99: 12795-12800.

4. Aguda BD, Kim Y (2008) MicroRNA regulation of a cancer network: Consequences of the feedback loops involving miR-17-92, E2F, and Myc. Proc Natl Acad Sci U S A 105: 19678-19683.

5. Li Y, Li Y, Zhang H, Chen Y (2011) MicroRNA-mediated Positive Feedback Loop and Optimized Bistable Switch in a Cancer Network Involving miR-17-92. PLoS ONE 6: e26302.

6. Hornung G, Barkai N (2008) Noise Propagation and Signaling Sensitivity in Biological Networks: A Role for Positive Feedback examples of interlinked positive and negative feedback loops. PLoS Comput Biol 4: 0055-0061.

7. Simpson ML, Cox CD, Sayler GS (2003) Frequency domain analysis of noise in autoregulated gene circuits. Proc Natl Acad Sci U S A 100: 4551-4556.

8. Papoulis A *Probability, Random Variables and Stochastic Process* (McGrawHill, New York, 1991).

9. Hooshangi S, Thiberge S, Weiss R (2005) Ultrasensitivity and noise propagation in a synthetic transcriptional cascade. Proc Natl Acad Sci U S A 102: 3581-3586.
