# Supplementary material for: Noise Propagation in Gene Regulation Networks Involving Interlinked Positive and Negative Feedback Loops
Source: PLoS One. 2012 Dec 20;7(12):e51840. doi: 10.1371/journal.pone.0051840 (PMC3527455; doi:10.1371/journal.pone.0051840)
Supplement: Text S2 — Effects of the positive feedback loop and miRNA inhibition on noise amplification and the sensitivity of the miRNA module when the initial steady is in the on-state or the off-state in a bistable region. (PDF) [file pone.0051840.s002.pdf]

Noise propagation in gene regulation networks involving interlinked positive and negative feedback loops (*Supplementary Text S2*)

Hui Zhang, Yueling Chen, Yong Chen*

∗ E-mail: ychen@lzu.edu.cn

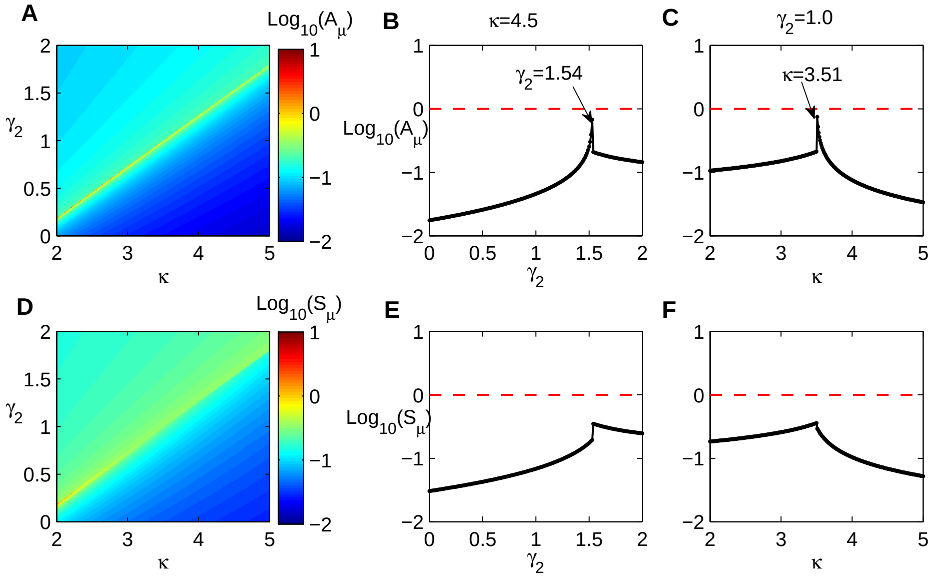

Figure 1. Effect of the positive feedback κ and miRNA inhibition γ_2 on the miRNA in the case of the initial steady on-state. (A) The noise amplification A_μ and (D) the sensitivity S_μ of miRNAs as a function of κ and γ_2 when the initial stable steady-state of the system is on-state in the bistable region. A_μ and S_μ for $\kappa = 4.5$ (B, E) and $\gamma_2 = 1.0$ (C, F), respectively. A_μ and S_μ reach the maximal values at $\gamma_2 = 1.54$ (B, E) and $\kappa = 3.51$ (C, F), respectively. Note that A_μ is always less than 1. Parameters are $\alpha = 0.15$, $\gamma_1 = 1.0$, $\varepsilon = 0.05$, $\tau_0 = 0.5$.

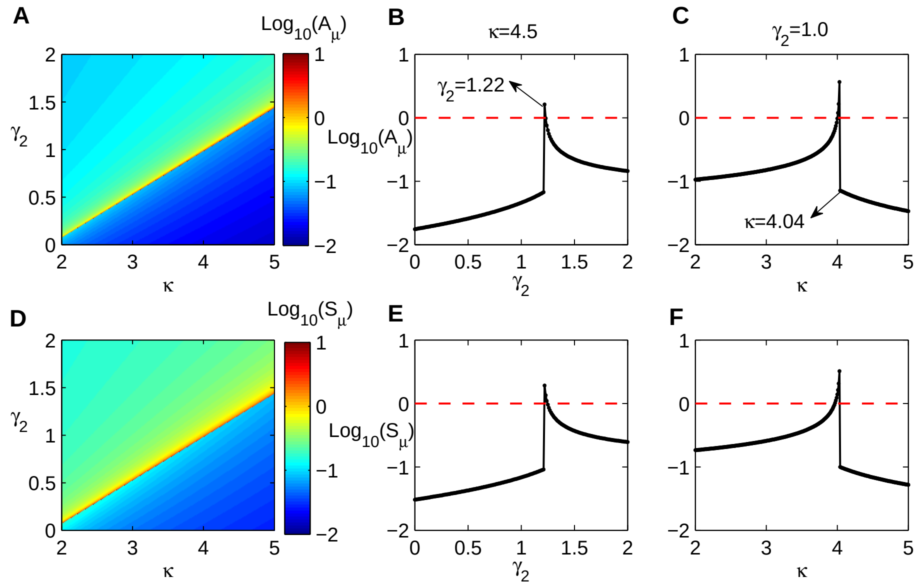

Figure 2. Effect of the positive feedback κ and miRNA inhibition γ_2 on the miRNA in the case of the initial steady off-state. (A) The noise amplification A_μ and (D) the sensitivity S_μ of the miRNAs as a function of κ and γ_2 for the initial steady off-state in the bistable region. A_μ and S_μ for $\kappa = 4.5$ (B, E) and $\gamma_2 = 1.0$ (C, F). Here, A_μ and S_μ reach their maximal values, for $\gamma_2 = 1.22$ (B, E) and $\kappa = 4.04$ (C, F). Note that $A_\mu > 1$ only in the vicinity of the boundary saddle nodes. Parameters are $\alpha = 0.15$, $\gamma_1 = 1.0$, $\varepsilon = 0.05$, $\tau_0 = 0.5$.
