# Supplementary material for: Noise Propagation in Gene Regulation Networks Involving Interlinked Positive and Negative Feedback Loops
Source: PLoS One. 2012 Dec 20;7(12):e51840. doi: 10.1371/journal.pone.0051840 (PMC3527455; doi:10.1371/journal.pone.0051840)
Supplement: Text S3 — Effects of the noise autocorrelation time and the time scale of the protein reaction on signal sensitivity and noise amplification for the system with an initial steady in the on-state or the off-state in a bistable region and an on/off state in a monostable region. (PDF) [file pone.0051840.s003.pdf]

Noise propagation in gene regulation networks involving interlinked positive and negative feedback loops (*Supplementary Text S3*)

Hui Zhang, Yueling Chen, Yong Chen*

* E-mail: ychen@lzu.edu.cn

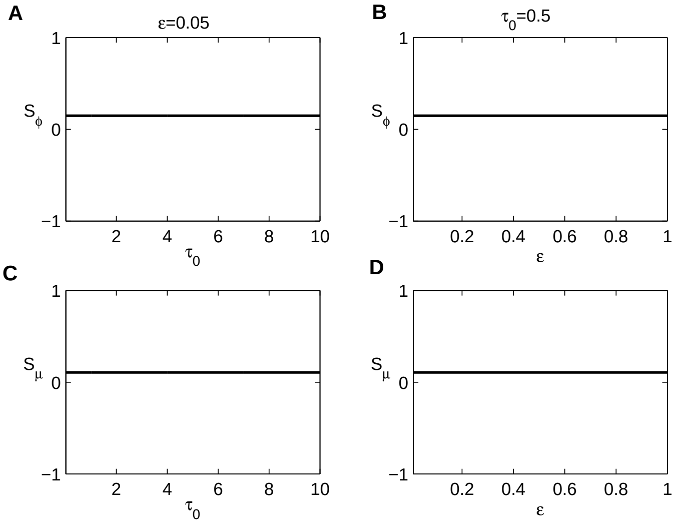

Figure 1. The role of the noise autocorrelation time τ_0 and the time scale of the protein reaction ε on signal sensitivity. The sensitivity of protein S_ϕ and miRNA S_μ as function of τ_0 (A, C) and ε (B, D), respectively. Parameters are $\alpha = 0.15$, $\gamma_1 = 1.0$, $\gamma_2 = 1.3$, $\kappa = 4.5$.

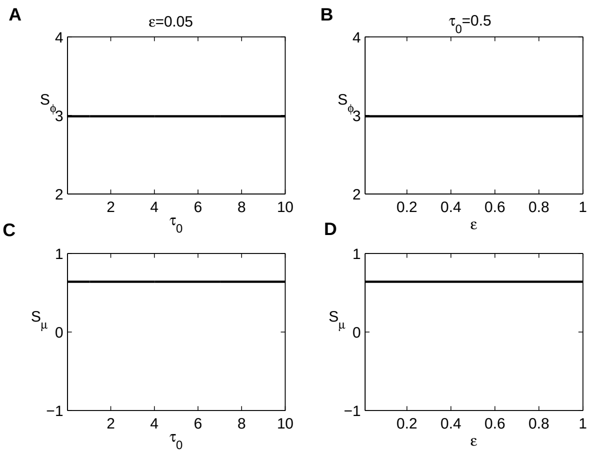

Figure 2. The role of the noise autocorrelation time τ_0 and the time scale of the protein reaction ε on signal sensitivity with initial steady *off-state* in bistable region. The sensitivity of protein S_ϕ and miRNA S_μ as function of τ_0 (A, C) and ε (B, D), respectively. Parameters are $\alpha = 0.15$, $\gamma_1 = 1.0$, $\gamma_2 = 1.3$, $\kappa = 4.5$.

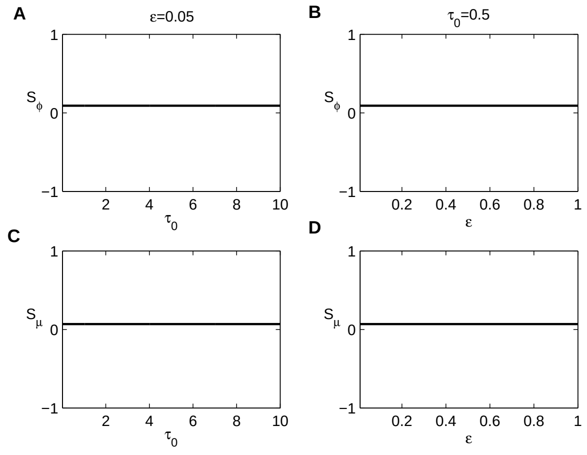

Figure 3. The role of the noise autocorrelation time τ_0 and the time scale of the protein reaction ε on signal sensitivity in monostable region with *on-state*. The sensitivity of protein S_ϕ and miRNA S_μ as function of τ_0 (A, C) and ε (B, D), respectively. Parameters are $\alpha = 0.15$, $\gamma_1 = 1.0$, $\gamma_2 = 1.0$, $\kappa = 4.5$.

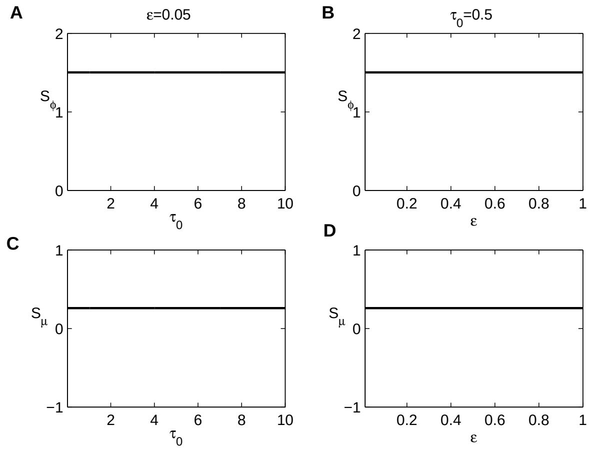

Figure 4. The role of the noise autocorrelation time τ_0 and the time scale of the protein reaction ε on signal sensitivity in monostable region with *off-state*. The sensitivity of protein S_ϕ and miRNA S_μ as function of τ_0 (A, C) and ε (B, D), respectively. Parameters are $\alpha = 0.15$, $\gamma_1 = 1.0$, $\gamma_2 = 1.9$, $\kappa = 4.5$.

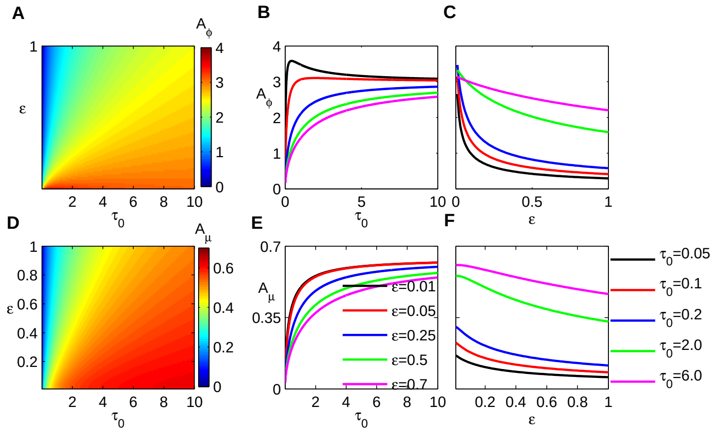

Figure 5. The role of the noise autocorrelation time τ_0 and the time scale of the protein reaction ε on noise amplification with initial steady *off-state* in bistable region. (A, D) The noise amplification in the protein module and miRNAs as a function of ε and τ_0. The noise amplification evolutes with τ_0 for different ε (B, E), and with ε for different τ_0 (C, F), respectively. Parameters are $\alpha = 0.15$,$\gamma_1 = 1.0$, $\gamma_2 = 1.3$, $\kappa = 4.5$.

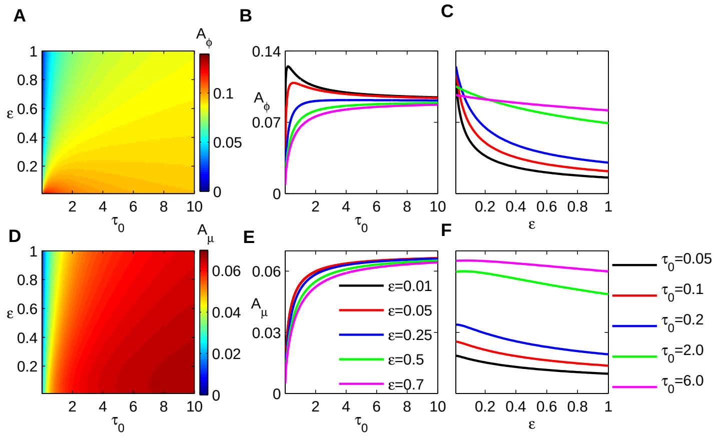

Figure 6. The role of the noise autocorrelation time τ_0 and the time scale of the protein reaction ε on noise amplification in monostable region with *on-state*. (A, D) The noise amplification in the protein module and miRNAs as a function of ε and τ_0. The noise amplification evolutes with τ_0 in input signal for different ε (B, E), and ε for different τ_0 (C, F), respectively. Parameters are $\alpha = 0.15$, $\gamma_1 = 1.0$, $\gamma_2 = 1.0$, $\kappa = 4.5$.

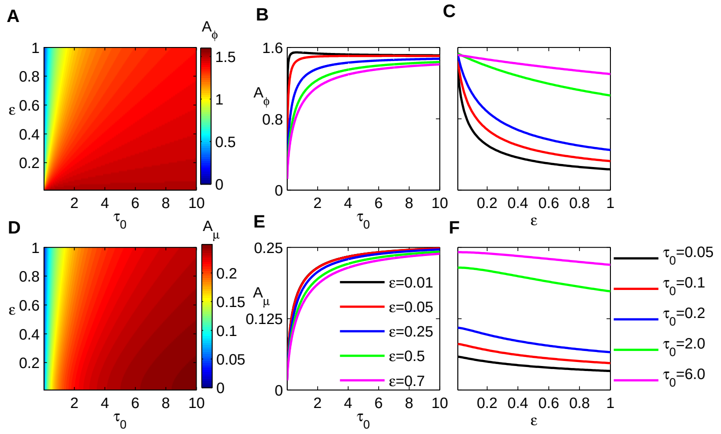

Figure 7. The role of the noise autocorrelation time τ_0 and the time scale of the protein reaction ε on noise amplification in monostable region with *off-state*. (A, D) The noise amplification in the protein module and miRNAs as a function of ε and τ_0. The noise amplification evolutes with τ_0 for different ε (B, E), and ε for different τ_0 (C, F), respectively. Parameters are $\alpha = 0.15$, $\gamma_1 = 1.0$, $\gamma_2 = 1.9$, $\kappa = 4.5$.
