# Supplementary material for: Noise Propagation in Gene Regulation Networks Involving Interlinked Positive and Negative Feedback Loops
Source: PLoS One. 2012 Dec 20;7(12):e51840. doi: 10.1371/journal.pone.0051840 (PMC3527455; doi:10.1371/journal.pone.0051840)
Supplement: Text S4 — The noise amplification from the stochastic simulation results with Gillespie algorithm and from the deduced results Equation (42) in Text S1. (PDF) [file pone.0051840.s004.pdf]

Noise propagation in gene regulation networks involving interlinked positive and negative feedback loops (*Supplementary Text S4*)

Hui Zhang, Yueling Chen, Yong Chen*

* E-mail: ychen@lzu.edu.cn

1 In comparison with the stochastic Gillespie simulation of the noise amplification.

The Langevin equation can provide insight into the the noise properties in biological systems. This Langevin approach should be statistically equivalent to the large-scale Monte Carlo simulation or Gillespie algorithm [1].

We used Gillespie's first reaction method [2, 3] to simulate extrinsic fluctuations. In our model, it includes two chemical species (E2F/Myc and miRNA) which participate in four chemical reactions: the production of protein E2F/Myc and miR-17-92, and their degradations. In addition, it exists an auto-catalytic growth of protein which is inhibited by miRNAs, and the protein can induce the transcription of miRNAs. The interaction between E2f/Myc and miR-17-92 is described in Figure 1 (or Figure 2B in main paper). p and m represent E2F/Myc and miRNAs, respectively.

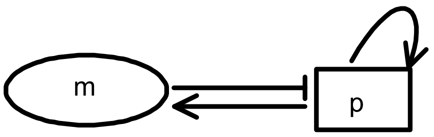

Figure 1. The model of the interaction between E2F/Myc (p) and miR-17-92 (m).

The model in Figure 1 is described the Equation [1-2] in main paper,

$$\frac{dp}{d\tau} = \alpha + \left(\frac{k_1 p^2}{\Gamma_1 + p^2 + \Gamma_2 m}\right) - \delta p \quad (1)$$

$$\frac{dm}{d\tau} = \beta + k_2 p - \gamma m. \quad (2)$$

The propensity function $W(i)$ (i represents the reaction i, $i = 1, 2, 3, 4$) of each chemical reaction i is

$$W = \left[\alpha + \frac{k_1 p^2}{\Gamma_1 + p^2 + \Gamma_2 m}, \quad \beta + k_2 p, \quad \delta p, \quad \gamma m\right]. \quad (3)$$

The formal steps for the Gillespie's algorithm are as follows,

1. Initialize the numbers of all species $p(0) = 100$ nM, $m(0) = 400$ nM, set time $t = 0$.
2. Calculate the propensity of each chemical reaction W_i, $i = 1, 2, 3, 4$.
3. Generate the uniform random numbers r_i, $i = 1, 2, 3, 4$.
4. For each reaction i, generate a putative next reaction time $\tau_i = -\ln(r_i)/W_i$, $i = 1, 2, 3, 4$.

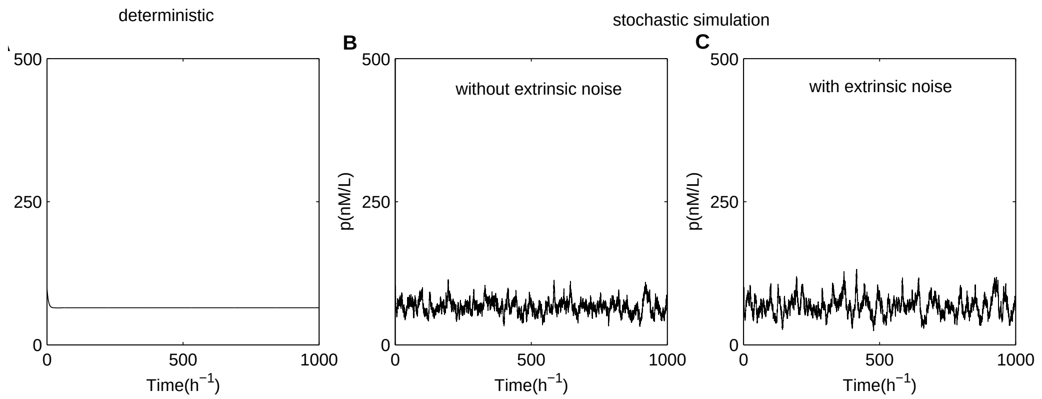

Figure 2. Comparison between the steady states of systems from the deterministic and stochastic simulations. The time trajectories from (A) the deterministic simulations and (B) the stochastic simulations without extrinsic noise and (C) with extrinsic noise, respectively. Parameters are, $\alpha = 20$ nM/ML, $k_1 = 400$ nMh^{-1}, $\Gamma_1 = 10^5$ nM^2, $\Gamma_2 = 450$ nM, $\delta = 0.4$ h^{-1}, $\beta = 6.7$ nMh^{-1}, $k_2 = 0.02$ h^{-1}, $\gamma = 0.02$ h^{-1}, $p(0) = 100$ nM, $m(0) = 400$ nM. The extrinsic noise intensity is $D = 0.000025$ and $\tau_0 = 2.0$.

5. Let μ be the reaction time with minimum τ_i.
6. Let t_0 be the time for the next discontinuous change in a reaction rate. Let j be the reaction whose rate change. In our system, $j = 1$.
7. Check whether $t + \tau_\mu < t_0$. If YES, then update the states of the species according to the reaction μ and change t to $t + \tau_\mu$. If NO, then change the reaction rate of reaction j and change t to t_0. Go to step 2.

The extrinsic fluctuations $\xi(t)$ is generated by an Ornstein-Unlenbeck time series [4],

$$\frac{d\xi(t)}{dt} = -\frac{\xi(t)}{\tau_0} + \frac{g(t)}{\tau_0}, \tag{4}$$

where τ_0 is the autocorrelation time of the noise and $g(t)$ is a white noise source. We set the interval time $\delta_t = 0.001$ and achieve the colored noise by calculating 100 realizations of the algorithm with different initial values of ξ.

We compare the steady states from the deterministic simulations (Figure 2A) using the fourth-order Runge-Kutta scheme and that from the stochastic simulations with Gillespie algorithm (Figure 2B and C). It shows that the protein level without extrinsic noise from the stochastic simulation (Figure 2B) fluctuates around the deterministic steady state (Figure 2A). The extrinsic noise only enhance the fluctuation (Figure 2C).

Then, to simulate the intrinsic and extrinsic noise effects on protein, we set that α' is fluctuated by $\xi(t)$, a color noise with mean zero and variance D/τ_0. We consider two copies [$p_1(t)$ and $p_2(t)$] of protein E2F/Myc in the same cellular environment [3]. The contribution of the extrinsic noise is

$$\eta^2_{ext} = \frac{\langle p_1(t)p_2(t)\rangle - \langle p\rangle^2}{\langle p\rangle^2}. \tag{5}$$

Here, $\langle p_1\rangle = \langle p_2\rangle = \langle p\rangle$ that is the value of deterministic dynamics.

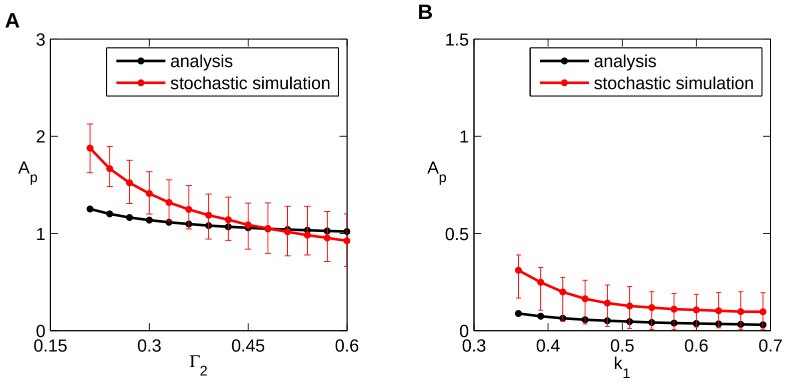

Figure 3. The comparison between the noise amplifications between from the stochastic simulations and from the Equation (42) in Text S1. (A) The system locates at off-state with $k_1 = 300$ nMh^{-1} and $\tau_0 = 3.5$. (B) The system is in on-state with $\Gamma_2 = 50$ nM and $\tau_0 = 2.0$. Other parameters are $\alpha = 20.0$ nML^{-1}, $\Gamma_1 = 10^5$ nM^2, $\delta = 0.4$ h^{-1}, $\beta = 6.7$ nMh^{-1}, $k_2 = 0.02$ h^{-1}, $\gamma = 0.02$ h^{-1}, $p(0) = 10$ nM, $m(0) = 40$ nM, and $D = 0.00002$.

In the similar way to deduce the noise amplification in Text S1. the elasticities H_{ij} are

$$H_{10} = -\frac{\langle\alpha\rangle}{\langle p\rangle\delta} \quad (6)$$

$$H_{11} = 1 - \frac{2k_1\langle p\rangle(\Gamma_1 + \Gamma_2\langle m\rangle)}{\delta(\Gamma_1 + \langle p\rangle^2 + \Gamma_2\langle m\rangle)^2} \quad (7)$$

$$H_{12} = -\frac{\Gamma_2 k_1\langle p\rangle\langle m\rangle}{\delta(\Gamma_1 + \langle p\rangle^2 + \Gamma_2\langle m\rangle)^2} \quad (8)$$

$$H_{20} = 0 \quad (9)$$

$$H_{21} = -\frac{\langle p\rangle}{\langle m\rangle}\frac{k_2}{\gamma} \quad (10)$$

$$H_{22} = 1. \quad (11)$$

Figure 3A presents that the the effects of negative feedback on extrinsic noise as the system at off-state between analytic results (black line) from the Equations (6)∼(11) and Equation (42) in Text S1 and the stochastic simulation results (red line). The simulations are the average of 100 trials and each simulation data is calculated from $7 * 10^7$ steps. It is shown that the noise amplification decreases with increasing the negative feedback level Γ_2. Similarly, Figure 3B plots that the comparison between the simulation results and the analytic results of the effect of the positive feedback on extrinsic noise with the on-state of the system. It is also observed that increasing the positive feedback level k_1 leads to the decrease of the noise amplification with $\Gamma_2 = 50$ nM. These stochastic simulations results are qualitatively consistent with the analytic results from the recipe presented in our work.

References

1. Ozbudak E M, Thattai M, Kurtser I, Grossman A D, van Oudenaarden A (2002) Regulation of noise in the expression of a single gene. Nat Genet 31: 69-73.

2. Gillespie D T (1976) A general method for numerically simulating the stochastic time evolution of coupled chemical reactions. J Comput Phys 22: 403-434.

3. Shahrezaei V, Ollivier J F, Swain P S (2008) Colored extrinsic fluctuations and stochastic gene expression. Mol Syst Biol 4: 196.

4. Fox R F, Gatland I R, Roy R, Vemuri G (1988) Fast, accurate algorithm for numerical simulation of exponentially correlated colored noise. Phys Rev A 38: 5938.
